# Global, regional, and national burden of vitamin A deficiency (1990–2021) and projections to 2042: associations with sociodemographic development

**DOI:** 10.3389/fnut.2025.1689903

**Published:** 2025-12-12

**Authors:** Ningjing Qin, Liwei Chen, Jin Guo, Jiabi Wang, Yujun He

**Affiliations:** 1 Department of Rehabilitation Medicine, Shenzhen Bao’an Shiyan People’s Hospital, Bao’an Clinical Institute of Shantou University Medical College, Shenzhen, China; 2 Department of Traditional Chinese Medicine, Taizhou Hospital of Zhejiang Province Affiliated to Wenzhou Medical University, Taizhou, China

**Keywords:** vitamin A deficiency, nutritional deficiencies, global burden of disease, predictive analysis, socio-demographic index

## Abstract

**Background:**

Vitamin A deficiency (VAD) remains a significant public health concern. Currently, there is a lack of comprehensive, up-to-date global assessments on the specific burden of VAD.

**Objective:**

This study aims to quantify the global, regional, and national disease burden attributable to VAD from 1990 to 2021, analyze its association with sociodemographic development, and project trends through 2042.

**Method:**

Using the Global Burden of Disease (GBD) 2021 database, we extracted data on deaths and disability-adjusted life years (DALYs) attributable to VAD across 204 countries and territories. We calculated absolute numbers, age-standardized rates (ASRs), estimated annual percentage changes (EAPCs), and relative changes of number and ASR at global, regional, socio-demographic index (SDI) quintile, and national levels. Pearson correlation and frontier analysis were used to examine the relationship between VAD burden and SDI. Bayesian age-period-cohort models were employed to project ASRs through 2042.

**Results:**

Between 1990 and 2021, the global number of deaths attributable to VAD decreased from 188458.416 to 17374.404; the ASR declined from 3.04 to 0.27 per 100,000 population (EAPC: −7.81%). Globally, VAD-attributable DALYs decreased from 18.79 million to 2.63 million, with the ASR falling from 303.72 to 40.10 per 100,000 population (EAPC: −6.70%). The largest reductions were observed in East Asia, Southeast Asia, and high-middle SDI regions, whereas the smallest reductions occurred in low SDI regions and Oceania. The burden consistently remained higher in males than in females, though this gap narrowed over time. Diarrheal diseases as the leading cause of VAD-attributable deaths. A strong negative correlation was identified between SDI and VAD burden; frontier analysis highlighted top-performing countries at equivalent SDI levels, which could serve as benchmarks. Projections indicated that VAD-attributable deaths and DALYs will continue to decline by 2042.

**Conclusion:**

Since 1990, global VAD-attributable mortality and DALYs have declined significantly, driven by socioeconomic development and public health interventions. However, substantial inequalities persist, with low SDI regions and males bearing a disproportionate residual burden. Sustained investment in targeted nutrition programs integrated with infectious disease control alongside broader socioeconomic development, particularly in the poorest settings, is necessary to accelerate progress toward eliminating VAD as a public health threat.

## 1 Introduction

Vitamin A deficiency (VAD) remains a pervasive nutritional disorder on the global scale, with the highest burden concentrated in low- and middle-income countries and other high-risk sub-populations. Retinol and its biologically active metabolites are indispensable for phototransduction, immunomodulation, cellular differentiation, and embryonic morphogenesis; consequently, inadequate vitamin A status precipitates a spectrum of pathologies, including night blindness, xerophthalmia, compromised immune competence, and growth faltering in early life (1, 2). A substantial body of epidemiological evidence has further linked VAD to heightened susceptibility to recurrent respiratory tract infections (1), iron-deficiency anemia (3), and adverse obstetric outcomes (4). Although mandatory fortification and targeted supplementation programs have been introduced in several nations, the prevalence of deficiency among pregnant women, young children, and individuals with chronic diseases continues to exceed acceptable thresholds (4, 5).

At present, a limited corpus of investigations has harnessed the Global Burden of Disease Study (GBD) 2021 repository to delineate regional heterogeneity in micronutrient deficiencies among children (6), adolescent (7), geriatric cohorts (8) and even its impacts observed during the COVID-19 pandemic (9); however, these analyses did not undertake a dedicated the burden specifically attributable to VAD. Among the extant literature, only the study by Zhao et al. (10) and Safiri et al. (11) have explicitly focused on VAD. Zhao et al. (10) confined their inquiry to interregional comparisons, whereas Safiri et al. (11) restricted their analytical scope to the Middle East and North Africa. Critically, both investigations relied on the preceding GBD 2019 iteration, and there is a lack of burden for diseases caused by VAD, as well as supplementary burden for mortality. Consequently, a comprehensive assessment of the global disease burden imposed by VAD, leveraging the most recent GBD 2021 data, remains conspicuously absent.

Between 1990 and 2021, the global landscape of micronutrient malnutrition underwent substantial transformation, characterized by marked achievements alongside enduring challenges. A precise characterization of the evolving disease burden attributable to VAD is therefore essential to inform the design and prioritization of targeted prevention and intervention strategies. In this context, the present study was undertaken to quantify the global burden of VAD from 1990 to 2021 and to forecast its trajectory over the ensuing two decades. The resulting evidence is anticipated to deepen the understanding of VAD-related disease burden, guide evidence-based policy formulation aimed at VAD reduction, and ultimately contribute to the global endeavor to mitigate this preventable public health threat.

## 2 Materials and methods

### 2.1 Detailed attribution logic of disease burden related to VAD

The quantification of VAD-related deaths and DALYs in this study was based on the Comparative Risk Assessment (CRA) framework of the GBD study. The core of this framework is to quantify the additional health losses caused by VAD by comparing differences in health outcomes across different exposure levels. The specific attribution process and methods are described as follows:

#### 2.1.1 Application of the comparative risk assessment framework

The CRA framework achieves attribution through two steps. Firstly, it identifies the “theoretical minimum risk exposure level” for VAD. Secondly, it quantifies the health losses corresponding to the risk difference between the actual exposure level and this minimum level, and ultimately attributes these losses to VAD. This framework ensures systematic quantification of the causal association between risk factors and health outcomes, rather than simple inference of correlations (12).

#### 2.1.2 Rationale for selecting risk-outcome pairs

The VAD-related risk-outcome pairs included in this study (associations between VAD and diarrhea/measles, and DALYs with VAD as the outcome) were all based on pre-defined, systematically reviewed and validated association sets from GBD 2021. The selection criteria were: (1) Sufficient epidemiological evidence confirming a causal relationship (e.g., VAD increases the severity and mortality risk of diarrhea and measles by impairing mucosal immunity); (2) Outcomes must meet the “attributability” criterion, meaning that reduced exposure to VAD directly lowers the probability or severity of the outcome; (3) Exclusion of associations dominated by confounding factors (e.g., indirect associations or outcomes with inconsistent evidence) (13).

#### 2.1.3 Definition and application of the theoretical minimum risk exposure level

The theoretical minimum risk exposure level for VAD is defined as a serum retinol concentration ≥0.7 μmol/L (corresponding to the exclusion criterion for ICD-10 codes E50–E50.9). This level is determined based on physiological thresholds: concentrations below this value lead to distinct pathological changes such as decreased immune function and epithelial cell damage, while concentrations ≥0.7 μmol/L minimize VAD-related health risks theoretically. In application, individuals with serum retinol concentration <0.7 μmol/L are classified as “exposed to VAD risk,” and their corresponding deaths and DALYs from diarrhea/measles in proportion to the risk difference (6).

#### 2.1.4 Role of the DisMod-MR 2.1 Bayesian meta-regression method

The DisMod-MR 2.1 tool was used to integrate heterogeneous global data and generate standardized epidemiological parameters through a Bayesian meta-regression model (14).

#### 2.1.5 Monte Carlo uncertainty propagation method

To quantify the uncertainty of attribution results, this study adopted the Monte Carlo simulation method. Multiple random samplings were performed on parameters generated by DisMod-MR 2.1, with each sampling based on the probability distribution characteristics of the parameters. Finally, 95% uncertainty intervals (UIs) for the number of deaths and DALYs were generated. This method captures the comprehensive impact of data heterogeneity, model assumption errors, and parameter estimation errors on the results, ensuring the robustness and reliability of the attribution outcomes. The core implication of the 95% UI is as follows: given the current data quality, model assumptions, and parameter estimation accuracy, there is a 95% statistical probability that the true disease burden value falls within this interval. The width of the 95% UI reflects the degree of uncertainty in the estimation results, a narrower interval indicates more sufficient data and more robust estimation, while a wider interval suggests sparse data or increased model extrapolation components, requiring more cautious interpretation of the results (15).

### 2.2 Research population and data compilation

Leveraging an integrative analytical architecture, GBD 2021 amalgamates heterogeneous data streams, employs the DisMod-MR 2.1 Bayesian meta-regression tool to generate internally consistent epidemiological parameters, and propagates uncertainty through probabilistic Monte Carlo simulations. This cohesive methodological suite yields standardized, cross-territorial estimates of population health loss (16). In the GBD 2021 framework, VAD is operationalized as a serum retinol concentration below 0.7 μmol/L, corresponding to the ICD-10 rubric E50–E50.9 and sequelae code E64.1. We quantified the population-level health loss attributable to VAD by examining two primary epidemiological metrics: deaths and disability-adjusted life-years (DALYs). The investigation encompassed 204 countries and territories, first stratified into 21 geographically contiguous GBD regions and subsequently assigned to five socio-demographic index (SDI) quintiles.

### 2.3 Data analysis

#### 2.3.1 Overview

Data analysis was initiated by evaluating the structural characteristics of the dataset, followed by the estimation of counts and rates for key metrics, including mortality and DALYs associated with VAD at the global, regional, and national levels. Subsequently, temporal variations in these measures from 1990 to 2021 were examined across diverse regions. This analytical process was applied to both case counts and age-standardized rates (ASRs), with the latter modeled using the equation: ln[ASR] = *a* + *bx* + *ε*, where *x* denotes the calendar year, and ASRs were expressed per 100,000 population. To quantify the relative changes (RC) over the period 1990–2021, the following formula was employed: Relative change (%) = [(Value in 2021 − Value in 1990)/Value in 1990] × 100%. This calculation was performed for both case counts and ASRs per 100,000 individuals (17). Furthermore, we additionally conducted an analysis of temporal, regional, and sex-specific variations in both the burden of VAD and the burden of other diseases attributable to VAD.

The estimated annual percentage change (EAPC) served to quantify temporal trends in ASRs, derived from a generalized linear model under the Gaussian distribution assumption (18). For EAPC calculation, calendar year was designated as the explanatory variable (*x*), with the natural logarithm of ASRs (ln[ASR]) as the dependent variable (*y*) for fitting the regression equation: *y* = *a* + *bx* + *ε*. EAPC was then calculated using the *β* parameter from the fitted regression line via the formula: EAPC = 100 × (exp(*β*) − 1) (19, 20). This computational framework is valid solely when ASR changes remain consistent across the entire observation period. Statistical hypothesis testing was conducted to evaluate the computed EAPC and account for random variability. Hypothesis testing for EAPC is equivalent to that for the slope of the fitted regression line; specifically, EAPC is considered valid if the slope demonstrates statistical significance. The testing process involved a *t*-test on the slope (*b*) of the fitted line, expressed as *tb* = *b*/*sb* (where *b* denotes the line slope and *sb* represents the standard error of *b*), with degrees of freedom (*V*) equal to the number of calendar years minus 2. Given that the standard error of slope b affects both the fitted line slope and EAPC, 95% confidence intervals (CIs) for EAPC were computed following the methodologies outlined in references (21, 22).

#### 2.3.2 Relationship between VAD burden and SDI

The SDI is a composite metric crafted by GBD researchers to evaluate a region’s socioeconomic standing. It incorporates per capita income, educational attainment, and fertility rates into a consolidated statistic ranging from 0 to 1, denoting the socioeconomic vitality and developmental status of a region or nation. Higher SDI values indicate improved socioeconomic conditions and better health outcomes. SDI classifies regions into five quintiles: low (0–0.454743), lower-middle (0.454743–0.607679), middle (0.607679–0.689504), upper-middle (0.689504–0.805129), and high (0.805129–1). The association between VAD burden and SDI will be examined via Spearman correlation analysis (23). In this section, JD_GBDR (V2.22, Jingding Medical Technology Co., Ltd.) was used for the drawing of the figures.

#### 2.3.3 Frontier analysis

To reduce redundancy while maintaining technical rigor, we employed stochastic frontier analysis to quantify the discrepancy between observed disease burdens and their theoretically achievable benchmarks. A benchmark frontier was established using countries with the lowest burden at each SDI level. This approach diverges from traditional inequality metrics by identifying countries whose disease burdens deviate upward from the frontier within homogeneous SDI strata, signaling potential systemic inefficiencies in healthcare delivery or suboptimal risk factor control. Such a framework explicitly facilitates policy learning among SDI-matched peers and disentangles macroeconomic development from intrinsic disease containment capacity, thereby generating actionable evidence for resource allocation that conventional cross-national comparisons cannot provide. These leading regions subsequently functioned as benchmarks and targets for other regions. For each country and territory, we calculated the “effective difference,” which represents the gap between the current VAD burden and its potential burden, adjusted for SDI (24). The methodological basis for the frontier analysis draws on the approach described by Guan et al. (25). Here are the specific steps: Selection of analytical methods: The Free Disposal Hull (FDH) method combined with Data Envelopment Analysis (DEA) was employed to plot the non-linear frontier. As a non-parametric approach, the FDH method allows relaxing the convexity assumption when defining the production possibility set, thereby providing greater flexibility in data processing (26). Data processing: Data were extracted from the GBD database, and 500 bootstrap samples were used to calculate the average disease burden value for each SDI score. The bootstrap method effectively evaluates the uncertainty and variability of data (24). Frontier smoothing: Locally Weighted Scatterplot Smoothing (LOESS) was applied to smooth the frontier, with a polynomial degree of 1 and a span of 0.2. As a non-parametric regression technique, LOESS can adapt to complex non-linear relationships and reduce noise through locally weighted regression. When plotting the frontier, countries with ultra-high efficiency were excluded to avoid the impact of outliers on the results (27). Frontier generation: A smoothed frontier was generated through the aforementioned steps, representing the achievable optimal level of health indicators across different SDI levels. Points on the frontier indicate the theoretically attainable optimal health performance under a given SDI (28). In this section, the R software package (version 4.2.3) and JD_GBDR (V2.22, Jingding Medical Technology Co., Ltd.) were utilized for figure generation.

#### 2.3.4 Predictive analysis

To predict the future trends of VAD burden over the next 20 years, we employed the Autoregressive Integrated Moving Average (ARIMA) model. This model utilizes the autocorrelation of time series data to predict future values based on historical observations. The core principle of ARIMA is that the data sequence is a time-varying random variable characterized by an autocorrelated structure. The ARIMA forecasting and the calculation of its 95% confidence intervals were performed with reference to “Forecasting: Principles and Practice” (2nd Edition) (29). For graphical visualization in this section, the R software package (version 4.2.3) and JD_GBDR (V2.22, Jingding Medical Technology Co., Ltd.) were employed.

## 3 Results

### 3.1 Overview of the global burden

#### 3.1.1 Results of the global and regional trend analysis for VAD

From 1990 to 2021, the global burden of VAD showed a significant downward trend in both mortality and DALYs.

##### 3.1.1.1 VAD-related mortality (1990–2021)

Globally, VAD-attributable deaths decreased significantly, from 188458.42 (95% UI, −510559.55–756085.57) in 1990 to 17374.40 (95% UI, −56819.02–75974.93) in 2021, with a relative change (RC) of-90.78%.

The ASR declined from 3.04 per 100,000 (95% UI: −8.23–12.17) in 1990 to 0.27 per 100,000 (95% UI: −0.89–1.19) in 2021, with an RC of −91.06% and an estimated annual percentage change (EAPC) of −7.81 (95% CI, −8.30, −7.31).

By sex, both males and females saw decreases in deaths and ASRs, with females showing a slightly greater reduction: death RC (−91.37% vs. −90.31% in males) and ASR RC (−91.61% vs. −90.62% in males), along with a lower EAPC (−7.98, 95% CI: −8.52, −7.44 vs. -7.67, 95% CI: −8.13, −7.21 in males).

Across SDI groups, the high-middle SDI group had the largest reduction in death numbers (RC: −99.03%, EAPC: −12.96, 95% CI: −13.24, −12.68), while the low SDI group had the smallest (RC: −84.9%).

Regionally, East Asia had the largest decline in death numbers (RC: −99.74%, EAPC: −17.59, 95% CI: −18.15, −17.03), and Australasia the smallest (RC: −73.23%, EAPC: −0.51, 95% CI: −2.48, 1.49) (Supplementary Table S1).

##### 3.1.1.2 VAD-related DALYs (1990–2021)

Globally, VAD-attributable DALYs decreased from 18794772.31 (95% UI: −43428353.62–69158948.13) in 1990 to 2663755.67 (95% UI: −3931015.05–8118159.57) in 2021, with an RC of −85.83%.

The ASR declined from 303.72 per 100,000 (95% UI: −699.04–1114.52) in 1990 to 40.10 per 100,000 (95% UI: −62.87–125.13) in 2021, with an RC of −86.8% and an EAPC of −6.70 (95% CI: −7.09, −6.32).

By sex, females had a slightly greater reduction in DALY numbers (RC: −86.01% vs. −85.68% in males) and ASR (RC: −87.03% vs. −86.62% in males).

Across SDI groups, the low-middle SDI group showed the largest reduction in DALY numbers (RC: −92.11%, EAPC: −8.36, 95% CI: −8.73, −7.98), while the high SDI group had the smallest (RC: −80.03%).

Regionally, Southeast Asia exhibited the largest decline in DALY numbers (RC: −94.57%, EAPC: −9.14, 95% CI: −9.28, −8.99), and Oceania the smallest (RC: −61.58%, EAPC: −3.73, 95% CI: −4.26, −3.19) (Supplementary Table S2).

**Figure 1:**
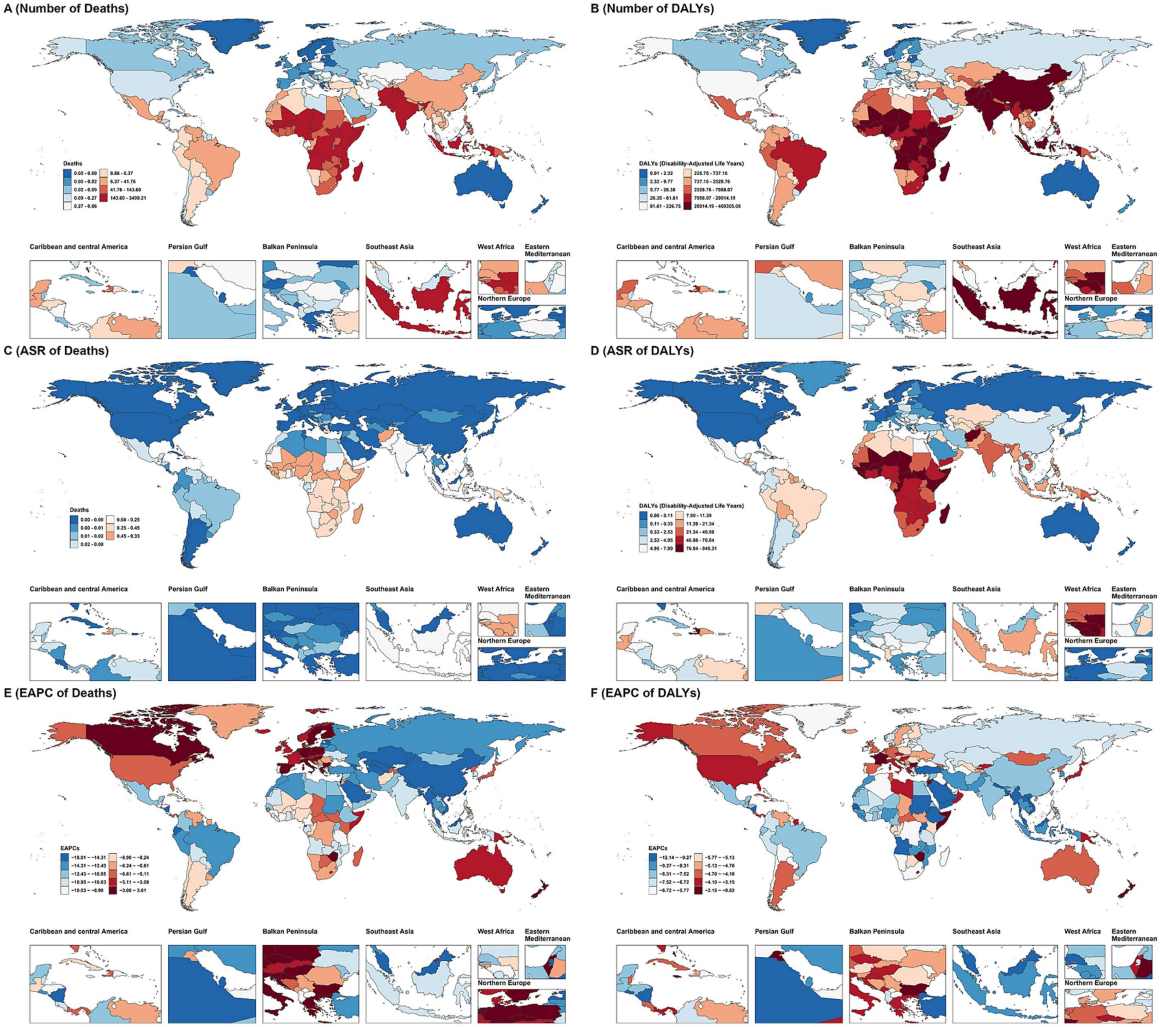
The number of deaths **(A)** and DALYs **(B)** VAD in 2021. The ASR of deaths **(C)** and DALYs **(D)** VAD in 2021. The EAPC of deaths **(E)** and DALYs **(F)** of VAD from 1990 to 2021.

The numbers of deaths and DALYs, along with ASR, for VAD in 2021, and EAPC, from 1990 to 2021 across 204 countries and territories globally are shown in Figure 1.

#### 3.1.2 Results of the trends by year, sex and regions for VAD

**Figure 2:**
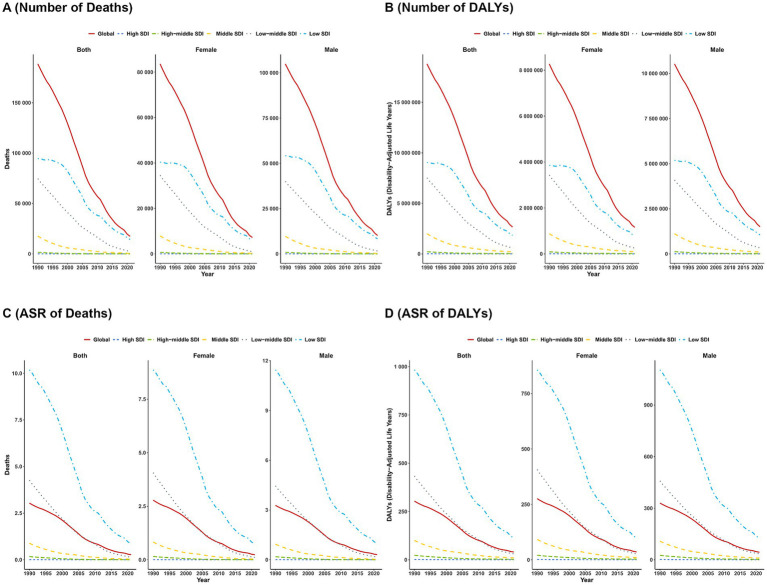
The trends of number in deaths **(A)** and DALYs **(B)**, and ASR in deaths **(C)** and DALYs **(D)** VAD categorized by global and five SDI regions from 1990 to 2021.

From 1990 to 2021, the global burden of VAD and that across all SDI-based regions decreased significantly across all sex and regions. Notably, the most pronounced reduction was observed in low SDI regions, whereas high SDI regions consistently maintained a low burden throughout the period. Despite the widespread downward trend in VAD burden across all regions, the burden in low SDI and low-middle SDI regions remained substantially higher than that in other regions (Figure 2).

**Figure 3:**
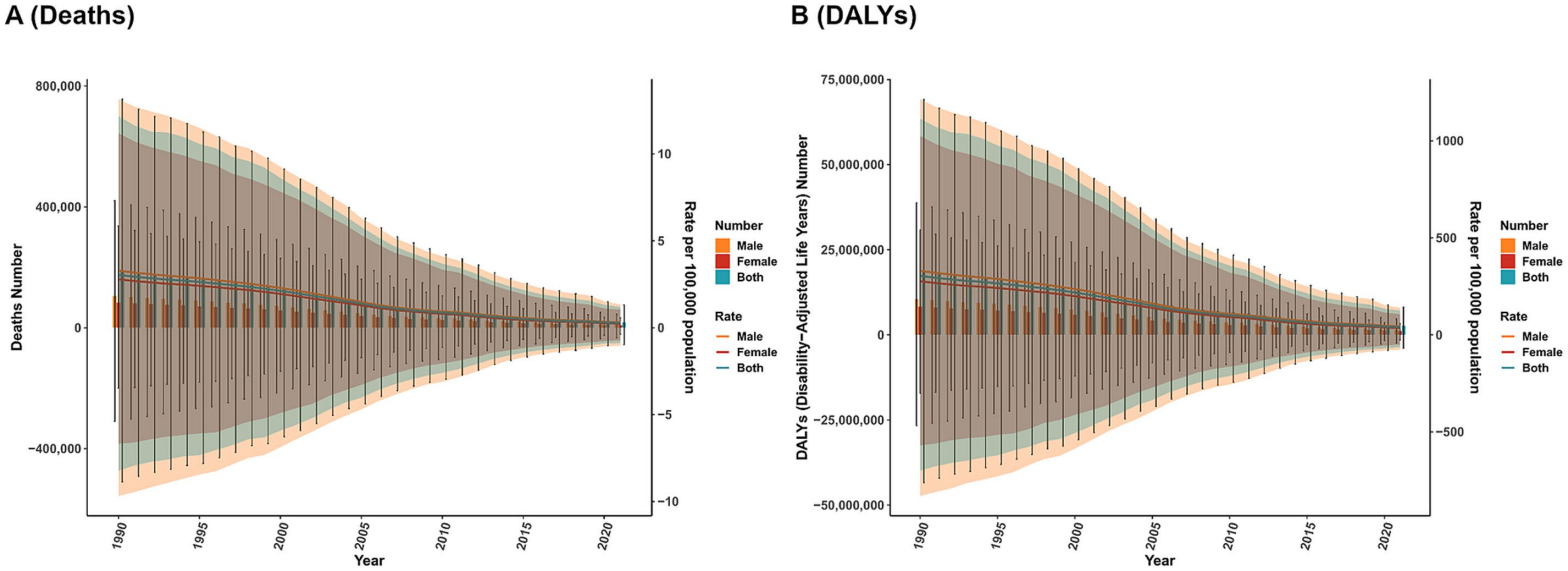
The trends of case number and ASR in deaths **(A)** and DALYs; **(B)** for VAD across different genders from 1990–2021. Shaded regions represent the predicted trend and its 95% UI.

From a gender perspective, both mortality and DALYs burdens attributed to VAD were consistently higher in males than in females. Nonetheless, such gender disparities have been gradually narrowing over the period from 1990 to 2021 (Figure 3).

#### 3.1.3 Results of the diverse disease burdens associated with VAD

**Figure 4:**
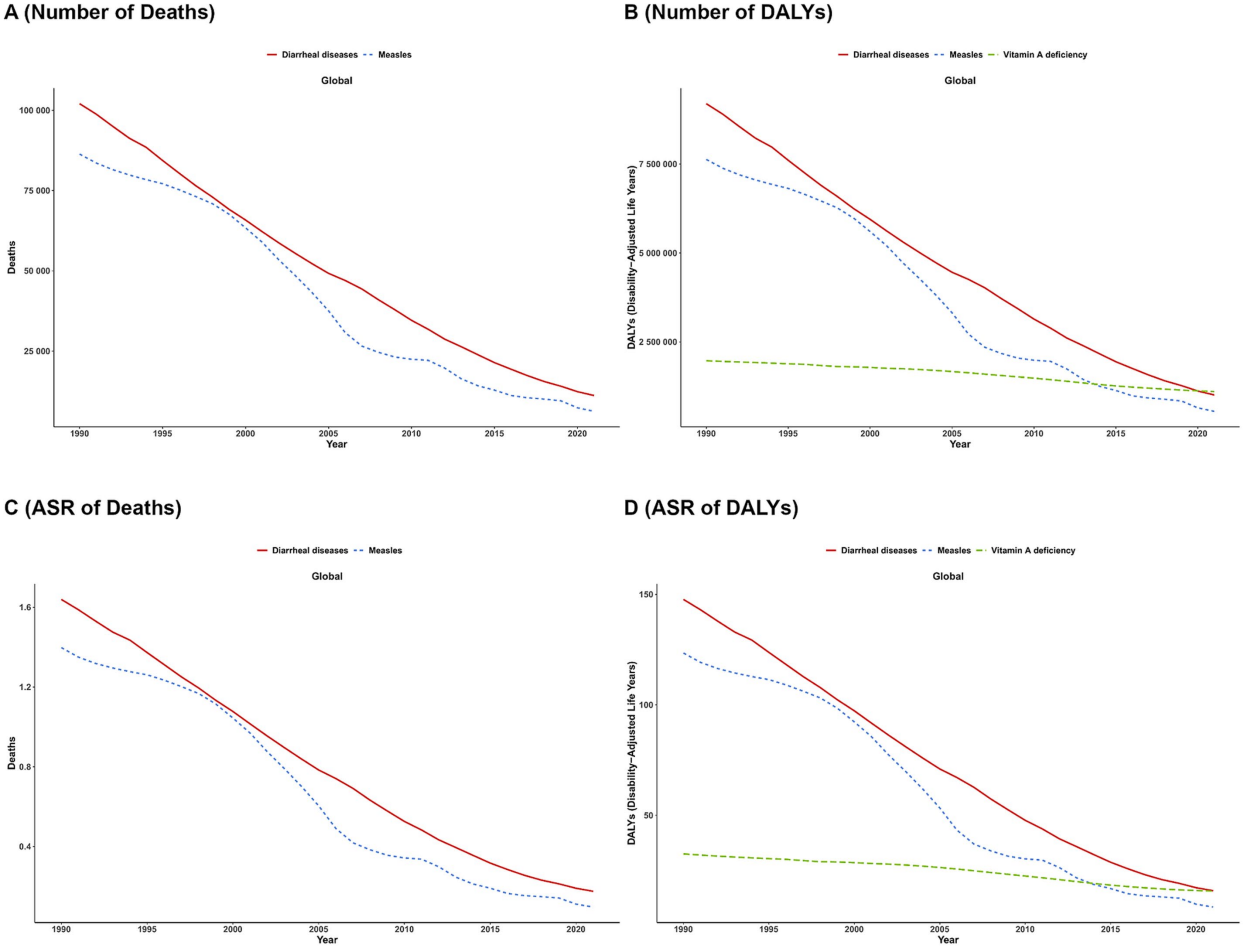
Temporal trends in VAD-attributable burden by cause, 1990–2021. **(A)** number of deaths; **(B)** number of DALYs; **(C)** ASR of deaths; **(D)** number of DALYs.

In the GBD 2021 database, VAD contributes to deaths from two diseases (diarrhea and measles), while it is responsible for DALYs associated with three diseases (diarrhea, measles, and VAD itself). Globally, the VAD-attributable burden of all these conditions exhibited a consistent declining trend from 1990 to 2021 (Figure 4).

**Figure 5:**
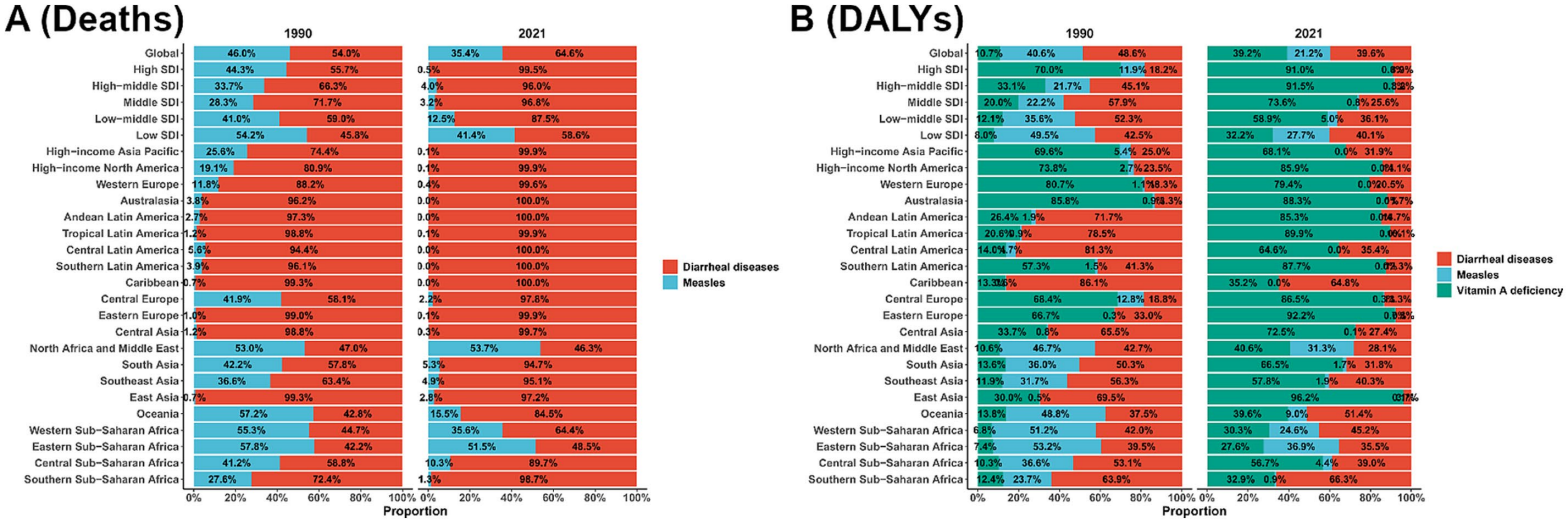
Temporal shifts in the etiological composition of VAD-attributable burden by region and SDI, 1990 vs. 2021. **(A)** deaths; **(B)** DALYs.

Figure 5A depicts the proportional distribution of VAD-attributable deaths from diarrheal disease (red) and measles (light blue) across global and regional strata (stratified by the SDI) in 1990 and 2021. Globally, diarrheal disease accounted for 54.0% of VAD-related deaths in 1990, versus 46.0% for measles; by 2021, diarrheal disease increased to 64.6%, while measles declined to 35.4%. In high-SDI regions, measles contributed 44.3% of such deaths in 1990 but plummeted to 0.5% in 2021, with diarrheal disease dominating (99.5%). In low-SDI regions, measles still accounted for 41.4% of VAD-attributable deaths in 2021, highlighting the persistent burden of infectious diseases. Figure 5B extends the analysis to include VAD (green), alongside diarrheal disease (red) and measles (blue). Globally, VAD represented 48.6% of VAD-attributable DALYs in 1990, followed by measles (21.7%) and diarrheal disease (29.7%). By 2021, the proportions of VAD (39.6%) and diarrheal disease (39.2%) converged, while measles remained stable at 21.2%. In high-SDI regions, VAD constituted 70.0% of DALYs in 1990 and rose to 91.0% in 2021, with both measles and diarrheal disease dropping below 10%. In low-SDI regions (e.g., Sub-Saharan Africa), measles (27.7%), diarrheal disease (40.1%), and VAD (32.2%) continued to collectively drive the major burden in 2021. Across regions, diarrheal disease remained a dominant contributor to both deaths and DALYs in low-SDI settings, with VAD persisting as a critical issue. In contrast, the burden of measles declined sharply in high-SDI regions, reflecting reduced susceptibility to infectious etiologies.

**Figure 6:**
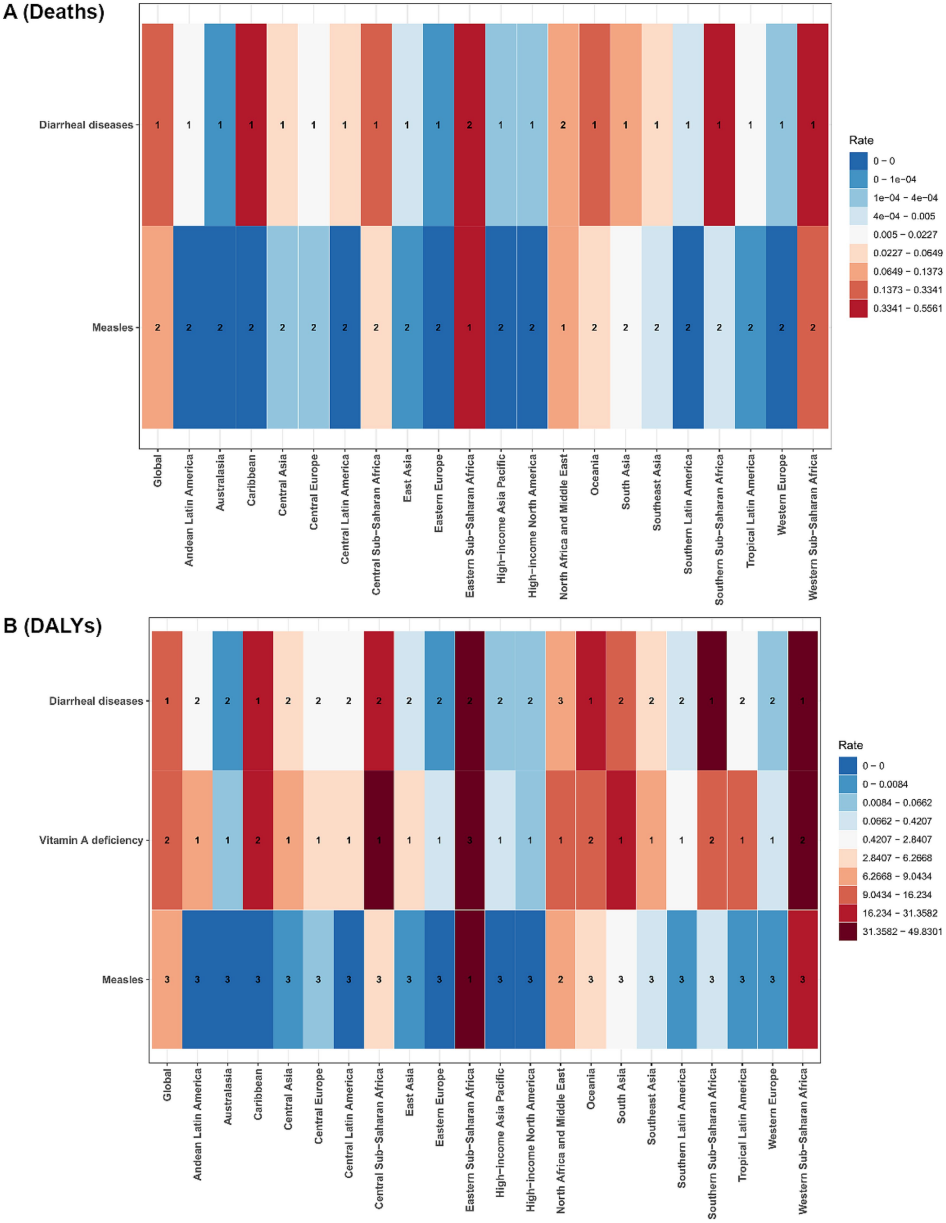
Regional ranking of rate for VAD-attributable conditions, by burden type. **(A)** deaths; **(B)** DALYs.

Figure 6A depicts the rate distribution of VAD-attributable deaths involving two diseases (diarrheal diseases and measles) across global regions, whereas Figure 6B illustrates the rate distribution of VAD-related DALYs for three conditions (diarrheal diseases, VAD, and measles). In the death burden (Figure 6A), measles exhibited predominantly low rates across most regions (e.g., Australasia, Western Europe), while diarrheal diseases showed higher rates in low-SDI regions such as Central and Eastern Sub-Saharan Africa. For DALYs (Figure 6B), VAD displayed striking regional variability: extremely high rates were observed in Central Asia and Sub-Saharan Africa, underscoring the persistent nutritional burden in these areas. In contrast, measles consistently showed low DALY rates globally, indicating its minimal contribution to disability. Diarrheal diseases, mirroring their death burden pattern, exhibited elevated DALY rates in regions like Tropical Latin America and Western Sub-Saharan Africa. Collectively, measles imposed negligible burdens in high-SDI regions across both outcomes, whereas diarrheal diseases (in both deaths and DALYs) and VAD (exclusively in DALYs) exerted substantial, region-specific burdens, particularly in low-SDI settings.

### 3.2 Results of the relationship between VAD burden and SDI

**Figure 7:**
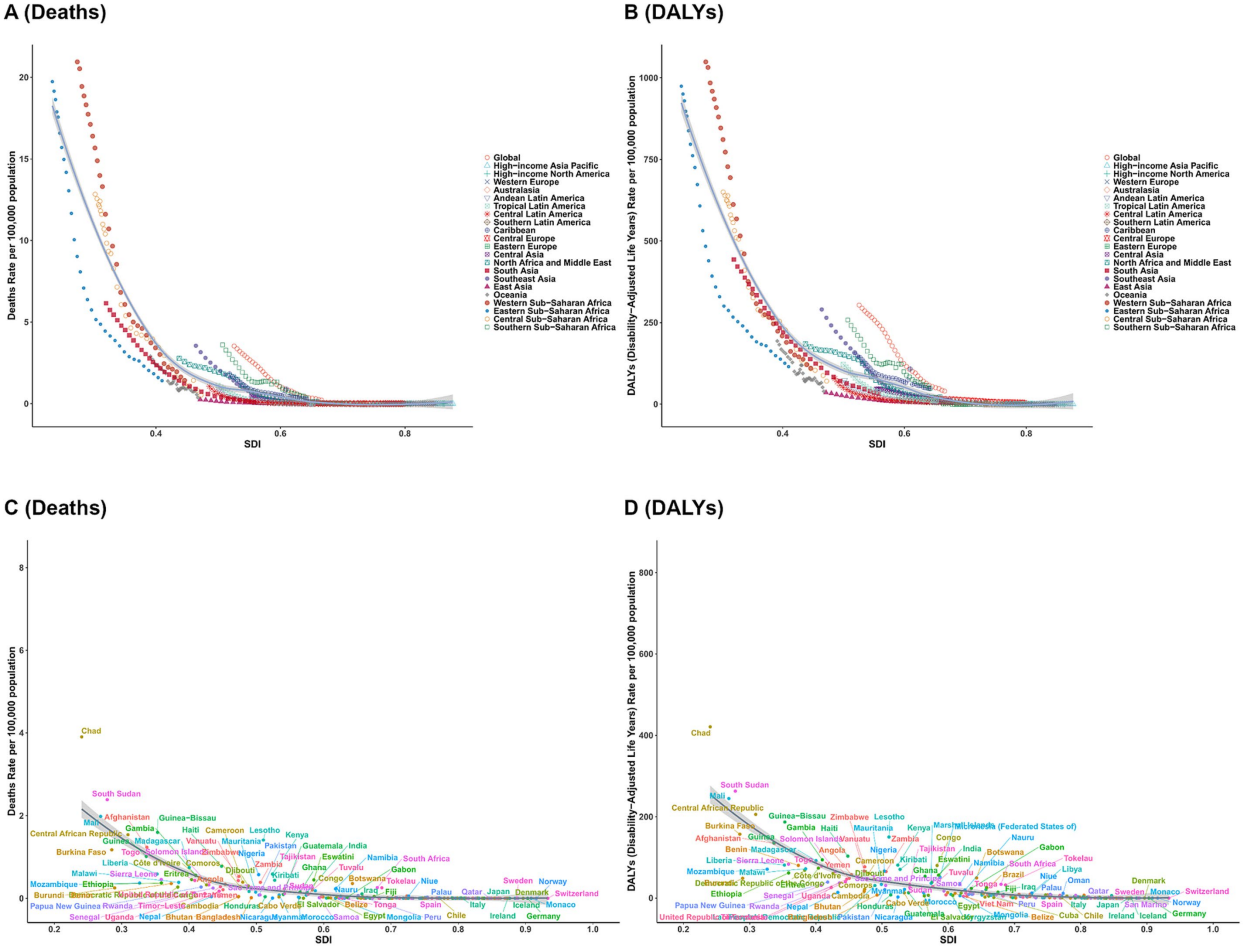
Relationship between SDI and the burden of VAD: results of deaths and DALYs across 21 GBD regions **(A)** deaths; **(B)** DALYs and 204 countries and territories. **(C)** deaths; **(D)** DALYs.

Figure 7 illustrates the association between the SDI and VAD-attributable disease burden. Across all panels, a robust inverse relationship emerged: as SDI increased (indicating higher socio-economic development), VAD-related death and DALYs rates declined sharply. At the regional scale (A, B), low-SDI regions (e.g., Western/Eastern Sub-Saharan Africa) exhibited the highest burdens, with death rates and DALYs often exceeding 15 per 100,000 population. In contrast, high-SDI regions (e.g., high-income North America, Western Europe) clustered near baseline rates, approaching zero. At the national level (C, D), this gradient was even more pronounced: low-SDI countries (e.g., Chad) showed extreme burdens, while high-SDI nations (e.g., the United States, most Western European countries) had near-negligible VAD-related mortality and DALYs.

### 3.3 Results of the frontier analysis for VAD

**Figure 8:**
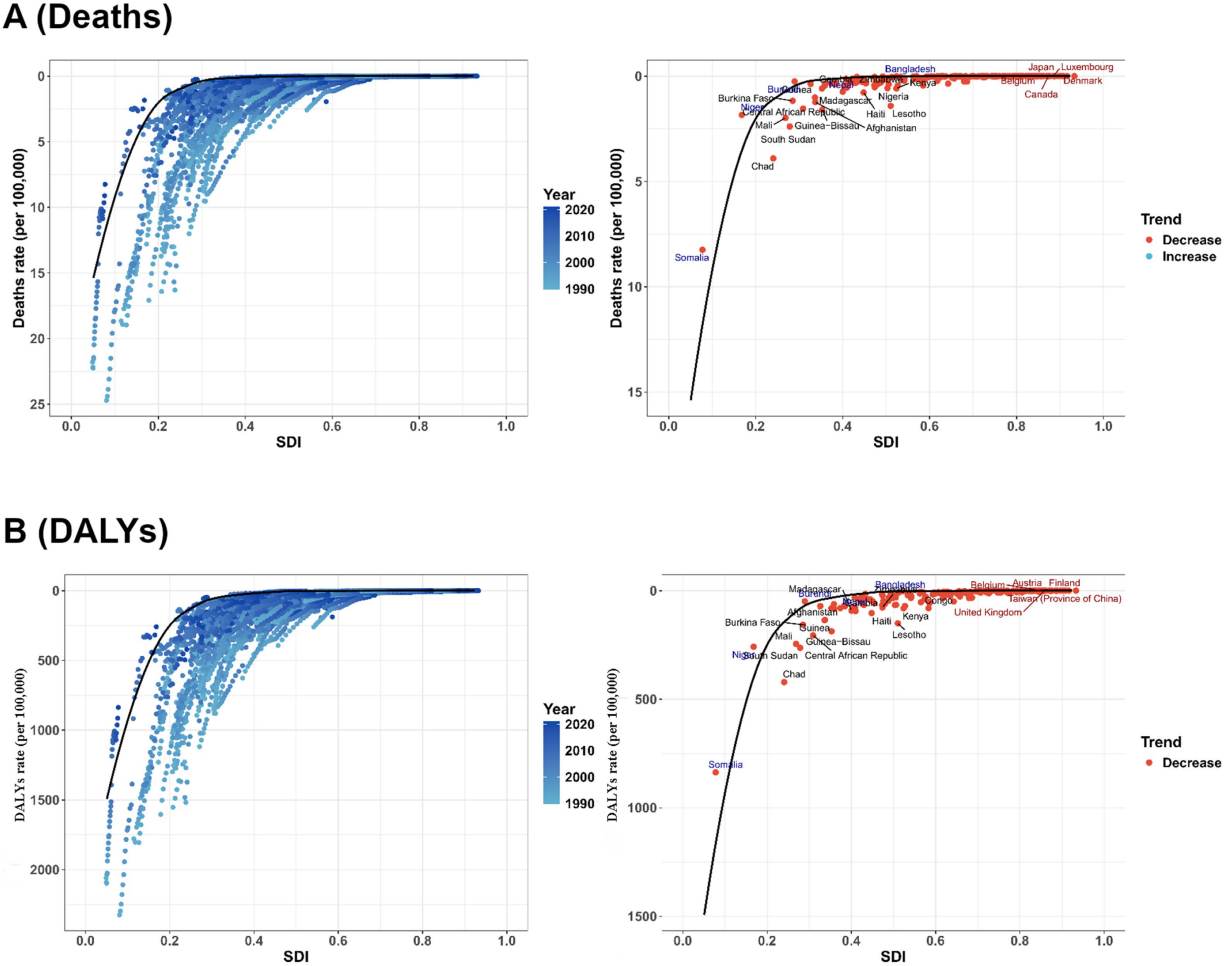
Frontier analysis, represented by the solid black line, examines the relationship between the SDI, the age-standardized rate (ASR), and deaths **(A)** as well as DALYs **(B)** in the context of VAD. The color gradients in left side subgraph show the change in years, with light colors representing 1990 and the darkest colors representing 2021. In right side subgraph, each dot represents a specific country or region in 2021. The top 15 countries with the largest deviations from the frontier are labeled in black text. Countries with a low SDI (>0.455) and the smallest deviations from the frontier are highlighted in blue text, while countries with a high SDI (>0.805) and significant deviations in terms of development level are highlighted in red text. The direction of change in the ASR from 1990 to 2021 is indicated by the color of the dots: red dots represent a decrease, whereas blue dots represent an increase.

Figure 8A (deaths) and Figure 8B (DALYs) depict the spatiotemporal dynamics of VAD-attributable disease burden relative to the SDI between 1990 and 2021. Across both outcomes, a strong inverse relationship emerged: as SDI increased (indicating higher socioeconomic development), VAD-related mortality and DALYs rates declined precipitously. Temporally, the color gradient in the left subpanels revealed consistent downward shifts, with the most pronounced reductions in low-SDI regions. The right subpanels further clarified national trends: nearly all countries exhibited a decreasing trend, though rare instances of marginal increases were observed.

Among countries with exemplary progress, high-SDI nations (e.g., Finland and Belgium) maintained extremely low VAD-attributable mortality and DALYs throughout the period. By 2021, their rates approached zero, reflecting successful control through socioeconomic advancement, robust healthcare systems, and targeted nutrition interventions.

In stark contrast, low-SDI countries (e.g., Chad and Central African Republic) exhibited profoundly high baseline burdens. Despite downward trends (red dots), these nations retained burdens far above the global average by 2021, highlighting persistent challenges in addressing nutritional inequities and healthcare access. Even with declining trends, their residual burden remains a critical public health issue, underscoring the need for intensified, context-specific interventions.

Collectively, these patterns demonstrate that while most countries have reduced VAD-associated health impacts, disparities persist: high-SDI nations have nearly eliminated the burden, whereas low-SDI settings despite progress continue to grapple with disproportionately high residual risk.

### 3.4 Results of the predictive analysis for VAD

**Figure 9:**
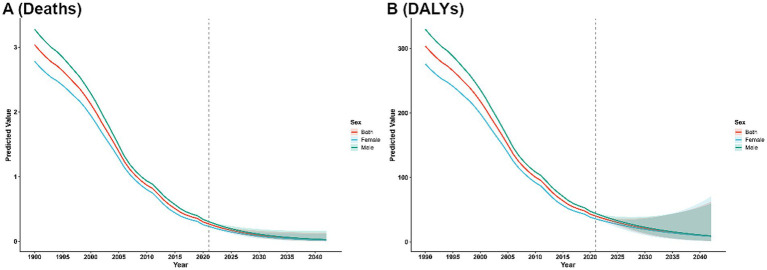
Predicted trends of VAD over the next 15 years (2022–2040). **(A)** ASR of deaths. **(B)** ASR of DALYs. Shaded regions represent the predicted trend and its 95% CI.

The ARIMA model validation and fitting results are presented in Supplementary Table S3. Projection analyses spanning the next 20 years suggest that the burden of deaths and DALYs attributable to VAD will persist in declining, irrespective of the overall population or disaggregation by sex (males or females) (Figure 9).

## 4 Discussion

The global burden of VAD has undergone substantial changes over the past three decades, with our analysis revealing a marked reduction in VAD-attributable mortality and DALYs from 1990 to 2021, coupled with distinct disparities across regions, sociodemographic strata, and sexes. These findings not only reflect progress in addressing VAD but also highlight persistent challenges that demand targeted public health interventions.

### 4.1 Global decline in VAD burden: progress and drivers

The significant downward trends in global VAD-related mortality (RC = −90.78%) and DALYs (RC = −85.83%) from 1990 to 2021 align with broader global efforts to combat micronutrient deficiencies. The consistent annual reduction, as indicated by the EAPC for age-standardized deaths rates (−7.81) and DALYs rates (−6.70), underscores the scalability and impact of these interventions when implemented with sustained political commitment and resource allocation. This progress can likely be attributed to decades of coordinated interventions, including the core strategy recommended by the World Health Organization: vitamin A supplementation (VAS). For instance, in India, the cost of VAS per avoided DALY is merely $23–50 (30). In Sub-Saharan African countries such as Nigeria, Kenya, and Burkina Faso, scaled-up coverage of VAS continues to avert substantial DALYs (31). The decline in VAD stems not only from direct interventions but also benefits from broader socioeconomic advancements, including improved educational attainment, enhanced sanitation facilities, and reduced poverty. It is important to note that the SDI, which aggregates socioeconomic development indicators, exhibits a strong negative correlation with VAD burden; however, SDI itself is not a direct causal factor. Instead, it serves as a composite proxy for a suite of downstream factors (e.g., better access to vitamin A supplementation via improved healthcare systems, increased dietary diversity enabled by reduced poverty, or enhanced awareness of nutritional needs through education) that directly influence VAD prevalence and associated burden. These factors have promoted dietary diversity and mitigated the overall risk of micronutrient deficiencies (32, 33). In Pakistan, studies have revealed that VAD-related costs are higher in impoverished households, accounting for 1.44% of the gross domestic product, which underscores the indirect impact of economic development on nutritional security (32). These are consistent with previous studies (10, 11).

Nevertheless, the 95% UIs of VAD-related ASRs in certain low-burden regions exhibit a cross-zero phenomenon. This result does not indicate a “negative true burden” but rather reflects statistical uncertainty under scenarios of extremely low disease burden. For instance, VAD-related deaths in high SDI regions are extremely rare, approaching zero or even being zero. This leads to increased variability in model estimates based on limited data, resulting in the lower bound of the 95% UIs extending below zero. Essentially, this phenomenon is a combined consequence of “data sparsity” and “randomness of statistical estimation,” suggesting that the VAD burden in such regions has dropped to near the “theoretical minimum risk level.” However, the current data cannot fully rule out the possibility that the true burden is close to zero.

### 4.2 Disparities across regions and sociodemographic strata

Despite global progress, our findings reveal striking inequities in VAD burden across regions and SDI groups. East Asia and high-middle SDI regions achieved the most dramatic reductions in mortality (RC = −99.74% and −99.03%, respectively), while low SDI regions and Australasia showed the smallest declines (RC = −84.9% and −73.23%, respectively). Similarly, for DALYs, Southeast Asia (RC = −94.57%) and low-middle SDI regions (RC = −92.11%) outperformed Oceania (RC = −61.58%) and high SDI regions (RC = −80.03%). These disparities are rooted in differential access to resources, socioeconomic development, and the implementation of nutrition-specific interventions.

East Asia’s success, for example, coincides with rapid economic growth, expanded healthcare coverage, and targeted nutrition programs (e.g., China’s national micronutrient supplementation initiatives) (34–36). Southeast Asia has promoted the reduction of VAD burden by relying on the “Rice Fortification Program” (37). In contrast, low SDI regions, predominantly in Sub-Saharan Africa, face persistent barriers such as poverty, food insecurity, weak healthcare systems, and recurrent crises (e.g., conflict, climate shocks), which hinder the delivery and uptake of VAD interventions (38). The persistently high burden in low and low-middle SDI regions, even amid global declines, underscores that VAD remains a marker of structural inequities, with marginalized populations bearing the brunt of unmet nutritional needs.

### 4.3 Temporal trends, regional disparities, and implications of VAD-attributable disease burden

The consistent global decline in VAD-attributable deaths and DALYs from 1990 to 2021 reflects the cumulative impact of decades of public health and nutrition interventions, including widespread vitamin A supplementation programs, improved access to preventive care, and advancements in infectious disease control. The proportional distribution of deaths, with diarrheal disease as the primary contributor, highlights the differential effectiveness of targeted strategies: while measles vaccination campaigns have substantially reduced measles-related mortality (39), particularly in high-SDI regions where measles now accounts for negligible burden, diarrheal disease persists as a critical challenge, especially in low-SDI settings. This disparity underscores the interplay between nutritional status and infectious disease susceptibility, as VAD impairs mucosal immunity and increases diarrheal severity, perpetuating a cycle that remains difficult to break without comprehensive improvements in sanitation, healthcare access, and nutritional support (40). The convergence of VAD and diarrheal disease as leading contributors to VAD-attributable DALYs by 2021 further emphasizes the dual burden of primary deficiency and its sequelae, with regional hotspots in Central Asia and Sub-Saharan Africa signaling unmet needs for context-specific interventions. Notably, the dominance of VAD itself in high-SDI regions’ DALYs suggests that while infectious complications have been mitigated, subclinical deficiency persists, warranting renewed focus on dietary diversification and vulnerable population screening. Collectively, these findings reinforce the importance of sustained investment in integrated nutrition and infectious disease programs, with targeted efforts in low-SDI regions to address the root causes of disparity and achieve equitable progress in reducing VAD-related burden.

This study found that diarrhea has replaced measles as the leading cause of VAD-related deaths. The potential reason is that the substantial global increase in measles vaccine coverage has led to a rapid decline in the measles burden (41). Although the absolute number of diarrhea-related deaths has decreased, the impact of VAD on its severe disease risk persists. Additionally, the unchanged attribution algorithm of the GBD study rules out methodological interference, indicating that this change is a relative proportion shift caused by the faster decline rate of measles.

In high-SDI regions, the proportion of DALYs directly attributed to VAD is relatively high. This is because these regions have effectively controlled infectious sequelae such as measles and diarrhea, highlighting the burden proportion of VAD itself (e.g., chronic health impairment from subclinical deficiency). Furthermore, these regions are more sensitive to the diagnosis of subclinical VAD. This phenomenon does not reflect aggravated VAD, but rather a transformation in the disease burden spectrum, suggesting that VAD prevention and control in such regions should shift focus to subclinical population screening and dietary optimization.

### 4.4 Gender dynamics in VAD burden

Our analysis revealed that while VAD-related mortality and DALYs remained consistently higher in males than females, gender disparities narrowed over time, with females experiencing slightly greater reductions in burden (e.g., RC of deaths: −91.37% vs. −90.31% in males). These findings align with emerging evidence that gender-sensitive nutrition programs, such as targeted supplementation for women of reproductive age and girls, may be closing gaps in access to interventions (42). However, the persistence of higher male burden warrants further investigation. Biological factors, such as sex-specific differences in vitamin A metabolism or higher susceptibility to severe infections in males, could contribute (43), but sociocultural factors, including differential care-seeking behavior or prioritization of male children in resource allocation, may also play a role (44). Addressing these disparities requires gender-disaggregated data collection and interventions tailored to the specific needs of both sexes.

This study observed that VAD-related mortality and DALYs in males remained consistently higher than those in females, with the gender gap gradually narrowing. Previously hypothesized biological or sociocultural factors may be associated with this discrepancy, but this study could not verify the causality of these factors. Given that the analysis relied on aggregated mortality and DALY data from the GBD study, without incorporating individual-level biological indicators (e.g., serum retinol metabolic enzyme activity), behavioral data (e.g., dietary intake structure, hygiene habits), or sociological data (e.g., intra-household resource allocation patterns, gender differences in healthcare accessibility), it was impossible to clearly distinguish the independent effects and root causes of various factors. This unresolved mechanism underlying gender differences should be an important direction for future research. It is recommended that subsequent studies adopt prospective cohort designs combined with individual-level tests (e.g., dynamic monitoring of serum retinol) and questionnaires (e.g., dietary and medical-seeking behaviors) to further clarify the driving factors of the high VAD burden in males, thereby providing evidence for formulating gender-targeted VAD prevention and control strategies.

### 4.5 The role of sociodemographic development in VAD reduction

The strong negative correlation between the SDI and VAD burden underscores the pivotal role of socioeconomic development in ameliorating nutritional deficiencies. High-SDI countries, characterized by advanced educational attainment, robust healthcare infrastructure, and enhanced food security, have nearly eliminated VAD-attributable mortality and DALYs, with such rates approaching zero by 2021, exemplified by nations including Belgium, Finland, and Canada. This aligns with the understanding that SDI encompasses not only economic resources but also access to education (which improves awareness of nutritional needs), healthcare (including preventive services), and stable food systems. As a composite metric integrating per capita income, educational attainment, and fertility rates, SDI acts as a proxy for a range of interrelated drivers that directly mitigate VAD: for example, higher educational attainment improves awareness of nutritional needs, robust healthcare infrastructure expands access to vitamin A supplementation, and stable food systems increase availability of vitamin A-rich foods. The observed correlation between SDI and VAD burden thus reflects the cumulative impact of these downstream causal factors, with SDI serving as a convenient summary measure rather than an independent cause of VAD reduction.

Conversely, despite downward trends, low-SDI countries (e.g., Chad, Central African Republic) continue to bear a disproportionately high residual burden, reflecting the challenges of addressing VAD in contexts marked by limited resources, weak governance, and high disease burdens. Notably, although some low-SDI countries (e.g., Niger, Burundi) have an overall low SDI, their VAD burden is close to the frontier level. Niger integrated vitamin A supplementation into the National Immunization Days in 1997 and subsequently pioneered the “National Vitamin A Supplementation Day” in 1999, ensuring that children aged 6–59 months receive two annual doses of vitamin A through mobilization activities (45). Burundi has focused on maternal and infant populations, providing vitamin A supplementation to pregnant women during prenatal care and offering dietary guidance to mothers of newborns through postnatal follow-up (46). In addition, although Egypt and Sudan belong to low-middle SDI regions, both have implemented corresponding effective public health policies: Egypt adopted a mandatory wheat flour fortification policy (since 2008) (47), while Sudan launched an integrated “VAS + diarrhea prevention and control” program in stable areas of the south (48). Findings from frontier analysis further emphasize that progress has been uneven: while most countries have moved toward reducing VAD burden, substantial disparities persist between high- and low-SDI settings. Nevertheless, low-SDI regions, such as Niger and Burundi, lie close to the frontier line, indicating a relatively low burden of VAD. This positions them as valuable models worthy of emulation by other countries with comparable SDI levels. This highlights the necessity of equitable investments in nutritional and healthcare systems within the world’s poorest regions.

### 4.6 Projections and future challenges

Our predictive analysis indicates that deaths and DALYs attributable to VAD will continue to decline over the next 20 years, which is encouraging. However, to ensure the sustained reduction in VAD, strategies must prioritize the following: Expanding interventions (such as vitamin A supplementation or fortification) in low-SDI regions, with a focus on hard-to-reach populations. Integrating VAD control with infectious disease programs specifically, prioritizing measles vaccination in low-SDI regions and diarrhea management in high-SDI regions, to address synergistic health risks. Strengthening the exchange of management policies among countries, particularly for low-SDI regions to learn from nations with comparable SDI levels that have achieved favorable outcomes.

Yet, due to the cross-zero phenomenon of 95% UIs for VAD-related ASRs in some low-burden regions, this uncertainty propagates into the prediction model. Therefore, the trend results for these regions require cautious interpretation.

### 4.7 Policy recommendations

To effectively reduce the global burden of VAD and address persistent disparities, targeted policy interventions should prioritize scaling up evidence-based nutrition strategies in low and low-middle SDI regions, including expanding universal vitamin A supplementation, strengthening food fortification of staple foods (for instance, low-SDI regions may prioritize vitamin A supplementation (each dose costs $0.2–0.5, supported by international aid and government sharing); middle-SDI regions can extend wheat flour fortification to edible oil (adding $0.1 worth of fortificants per liter, with half subsidized by the government).), and promoting dietary diversification through agricultural initiatives that increase access to vitamin A-rich foods, while integrating VAD control with infectious disease management by coupling supplementation with measles vaccination campaigns and water, sanitation, and hygiene improvements to mitigate the contributions of diarrheal disease and measles, which remain significant in low-SDI settings. Additionally, strengthening health information systems in data-limited regions is critical to enhance surveillance of VAD prevalence and associated burdens, enabling timely resource allocation to high-need subpopulations, while implementing gender-sensitive policies to address residual gender disparities, such as targeted outreach for males with persistently higher burdens and supporting women of reproductive age through antenatal care, will further advance equity. Finally, sustaining long-term investment in socioeconomic development in low-SDI regions, including education, healthcare infrastructure, and poverty alleviation, is fundamental given the robust inverse relationship between SDI and VAD burden, as addressing structural determinants like educational attainment and food security will drive equitable and sustained reductions in VAD globally.

### 4.8 Limitations

It is crucial to acknowledge several limitations of this study. Firstly, the estimates reported in this study are derived from statistical modeling frameworks embedded within the GBD 2021 infrastructure, which employ causal inference methodologies to quantify the proportion of disease burden attributable to specific factors based on exposure-response dynamics. These modeling approaches are predicated on several key assumptions, including linearity within dose–response relationships, comprehensiveness of exposure datasets, and the omission of unmeasured confounders, factors that may introduce potential biases into the attribution outcomes. Contrary to direct observational datasets, the findings herein constitute statistical extrapolations rather than direct causal evidence. Consequently, the absolute counts of deaths and DALYs ascribed to VAD warrant cautious interpretation. Secondly, data derived from the GBD 2021 database may be compromised by heterogeneity in data collection practices across geographic regions. Notably, in resource-constrained settings, issues such as underascertainment, diagnostic misclassification, or data incompleteness may contribute to imprecise estimates of disease burden (17). In addition, although the GBD model is sophisticated, it relies on the quality and quantity of basic input data. However, data quality varies significantly across countries, which may lead to lower reliability of estimation results in data-scarce regions (usually low SDI regions). Furthermore, the prediction model assumes that past trends will continue linearly, which may fail to capture potential future disturbing factors (e.g., the impact of climate on food security, new conflicts, and setbacks in nutrition programs related to pandemics). Lastly, projections are based on current trends and may not account for unforeseen disruptions, such as pandemics or economic crises.

## 5 Conclusion

The global burden of VAD has declined substantially over the past three decades, driven by socioeconomic development and targeted public health interventions. However, significant disparities persist, with low SDI regions and males bearing a disproportionate burden. Sustained progress requires equitable investment in nutrition interventions, integration with infectious disease control, and strengthening of health systems in vulnerable regions. By addressing these inequities, we can move closer to the goal of eliminating VAD as a public health threat worldwide.

## Data Availability

Publicly available datasets were analyzed in this study. This data can be found at: https://vizhub.healthdata.org/gbd-results/.
