# Supplementary material for: Global, regional, and national burden of vitamin A deficiency (1990–2021) and projections to 2042: associations with sociodemographic development
Source: Front Nutr. 2025 Dec 12;12:1689903. doi: 10.3389/fnut.2025.1689903 (PMC12742204; doi:10.3389/fnut.2025.1689903)
Supplement: Supplementary file 1 [file Data_Sheet_1.pdf]

Table S1 The case number and ASR of deaths of vitamin A deficiency in 1990 and 2021, and its temporal trends from 1990 to 2021, categorized by global, SDI and 21 regions.

Location name	1990		2021		1990-2021		
	**Case number (95% UI)**	**ASR (95% UI)**	**Case number (95% UI)**	**ASR (95% UI)**	**EAPC (95% CI)**	**RC of numbers (%)**	**RC of ASR (%)**
Global	188458.416(-510559.546-756085.569)	3.038(-8.226-12.174)	17374.404(-56819.019-75974.928)	0.272(-0.886-1.189)	-7.81(-8.30,-7.31)	-90.78	-91.06
**Sex**							
Male	104883.479(-310153.676-420900.544)	3.277(-9.679-13.143)	10158.054(-34283.089-43939.399)	0.307(-1.035-1.331)	-7.67(-8.13,-7.21)	-90.31	-90.62
Female	83574.937(-200527.953-336505.813)	2.782(-6.678-11.194)	7216.350(-22214.749-32093.693)	0.233(-0.717-1.040)	-7.98(-8.52,-7.44)	-91.37	-91.61
**SDI**							
High SDI	34.047(-46.963-160.046)	0.006(-0.008-0.026)	1.143(-1.927-6.015)	0.000(-0.000-0.001)	-8.56(-9.73,-7.37)	-96.64	-95.97
High-middle SDI	1544.957(-3162.563-7265.462)	0.170(-0.350-0.801)	14.950(-25.514-74.984)	0.002(-0.004-0.012)	-12.96(-13.24,-12.68)	-99.03	-98.58
Middle SDI	17565.264(-45270.980-80699.402)	0.883(-2.277-4.059)	454.332(-856.994-2223.585)	0.028(-0.053-0.138)	-10.19(-10.55,-9.81)	-97.41	-96.83

Low-middle SDI	74637.051(-187363.972-309301.837)	4.266(-10.709-17.644)	2604.913(-4963.671-11755.624)	0.138(-0.263-0.624)	-10.29(-10.94,-9.63)	-96.51	-96.76
Low SDI	94584.801(-272207.095-370680.224)	10.184(-29.240-39.666)	14285.586(-50965.966-62585.665)	0.857(-3.058-3.752)	-8.03(-8.45,-7.61)	-84.9	-91.59
**Regions**							
Andean Latin America	284.677(-527.734-1393.780)	0.529(-0.981-2.590)	8.118(-10.752-42.065)	0.013(-0.018-0.069)	-11.93(-12.15,-11.70)	-97.15	-97.47
Australasia	0.009(-0.013-0.045)	0.000(-0.000-0.000)	0.002(-0.003-0.012)	0.000(-0.000-0.000)	-0.51(-2.48,1.49)	-73.23	-76.61
Caribbean	645.076(-1866.493-3242.344)	1.529(-4.427-7.665)	132.111(-229.600-633.984)	0.346(-0.600-1.657)	-4.44(-4.94,-3.94)	-79.52	-77.4
Central Asia	321.365(-691.023-1575.476)	0.340(-0.731-1.666)	34.185(-69.783-166.821)	0.034(-0.070-0.167)	-8.27(-8.74,-7.80)	-89.36	-89.91
Central Europe	61.921(-124.790-258.526)	0.071(-0.145-0.299)	2.817(-4.837-14.165)	0.005(-0.009-0.027)	-7.14(-8.37,-5.89)	-95.45	-92.44
Central Latin America	1752.542(-3647.813-8350.862)	0.749(-1.563-3.571)	48.444(-72.289-251.631)	0.025(-0.037-0.130)	-10.27(-10.72,-9.80)	-97.24	-96.66

Central Sub-Saharan Africa	7055.492(-24822.391-28799.308)	6.533(-22.839-26.538)	707.381(-3205.054-3634.060)	0.336(-1.523-1.725)	-8.52(-9.44,-7.60)	-89.97	-94.86
East Asia	3098.737(-8348.933-15573.156)	0.271(-0.729-1.360)	7.951(-15.634-43.140)	0.001(-0.003-0.007)	-17.59(-18.15,-17.03)	-99.74	-99.53
Eastern Europe	3.151(-5.126-16.091)	0.002(-0.003-0.010)	0.053(-0.092-0.289)	0.000(-0.000-0.000)	-12.25(-13.20,-11.29)	-98.31	-97.12
Eastern Sub-Saharan Africa	37666.119(-100373.955-148378.126)	10.160(-26.992-39.793)	5994.170(-18827.659-25924.743)	0.936(-2.941-4.050)	-7.79(-8.07,-7.50)	-84.09	-90.78
High-income Asia Pacific	1.499(-1.784-6.990)	0.002(-0.002-0.007)	0.061(-0.099-0.329)	0.000(-0.000-0.001)	-7.32(-7.76,-6.88)	-95.93	-93.34
High-income North America	0.749(-1.139-3.790)	0.000(-0.001-0.002)	0.178(-0.282-0.920)	0.000(-0.000-0.000)	-4.43(-5.34,-3.51)	-76.22	-73.59
North Africa and Middle East	9427.193(-20846.877-38750.036)	1.842(-4.069-7.563)	885.631(-1643.788-3846.908)	0.148(-0.275-0.646)	-9.52(-10.26,-8.78)	-90.61	-91.94
Oceania	190.265(-427.624-764.089)	1.859(-4.160-7.458)	51.678(-137.912-259.547)	0.260(-0.693-1.304)	-4.42(-5.09,-3.74)	-72.84	-85.99
South Asia	67308.453(-163321.209-277396.079)	4.288(-10.402-17.649)	1822.224(-2096.907-8395.188)	0.118(-0.136-0.544)	-10.39(-	-97.29	-97.24

					11.34,-9.43)		
Southeast Asia	16529.434(-47989.238-74926.062)	2.842(-8.249-12.883)	373.213(-713.844-1918.421)	0.068(-0.130-0.349)	-11.08(-11.35,-10.80)	-97.74	-97.61
Southern Latin America	31.265(-61.097-149.146)	0.062(-0.120-0.294)	1.272(-2.337-6.578)	0.003(-0.006-0.017)	-8.46(-8.77,-8.15)	-95.93	-94.68
Southern Sub-Saharan Africa	1889.409(-3938.546-8521.191)	2.508(-5.211-11.306)	290.697(-437.233-1396.366)	0.369(-0.556-1.775)	-5.42(-5.91,-4.92)	-84.61	-85.28
Tropical Latin America	1722.391(-4626.418-8292.224)	1.066(-2.863-5.131)	18.817(-33.677-98.831)	0.011(-0.020-0.059)	-14.01(-14.40,-13.62)	-98.91	-98.95
Western Europe	1.381(-2.276-6.907)	0.001(-0.001-0.003)	0.339(-0.545-1.705)	0.000(-0.000-0.001)	-2.35(-3.10,-1.58)	-75.42	-72.57
Western Sub-Saharan Africa	40467.288(-131686.719-161088.271)	11.031(-35.887-43.846)	6995.062(-28786.089-31317.479)	0.863(-3.573-3.868)	-8.35(-8.82,-7.88)	-82.71	-92.17

Abbreviations: ASR, age-standardized rate; RC, relative change; EAPC, estimated annual percentage change; SDI, sociodemographic index; UI, uncertainty interval; CI, confidence interval.

Table S2 The case number and ASR of DALYs of vitamin A deficiency in 1990 and 2021, and its temporal trends from 1990 to 2021, categorized by global, SDI and 21 regions.

Location name	1990		2021		1990-2021		
	**Case number (95% UI)**	**ASR (95% UI)**	**Case number (95% UI)**	**ASR (95% UI)**	**EAPC (95% CI)**	**RC of numbers (%)**	**RC of ASR (%)**
Global	18794772.313(-43428353.619-69158948.130)	303.717(-699.037-1114.515)	2663755.669(-3931015.048-8118159.572)	40.100(-62.874-125.130)	-6.70(-7.09, -6.32)	-85.83	-86.8
**Sex**							
Male	10524281.119(-26626902.271-38850511.248)	329.818(-829.913-1214.233)	1506966.819(-2480125.370-4673697.454)	44.146(-76.335-139.787)	-6.65(-7.02, -6.29)	-85.68	-86.62
Female	8270491.195(-17148451.570-30799926.272)	275.929(-569.904-1025.033)	1156788.850(-1466742.242-3331890.471)	35.780(-49.013-106.113)	-6.77(-7.18, -6.36)	-86.01	-87.03
**SDI**							
High SDI	12024.460(2257.291-24923.626)	1.885(0.299-3.981)	2401.012(1452.946-3533.757)	0.332(0.183-0.509)	-5.16(-5.63,	-80.03	-82.4

					-4.69)		
High-middle SDI	216707.617(-228883.211-741777.979)	23.483(-25.566-81.568)	27802.095(17385.119-39302.663)	2.976(1.755-4.357)	-6.89(-7.21,-6.56)	-87.17	-87.33
Middle SDI	1988518.312(-3788983.273-7716595.109)	100.201(-190.313-388.269)	199673.688(57728.139-377373.415)	10.062(1.522-20.957)	-7.16(-7.26,-7.06)	-89.96	-89.96
Low-middle SDI	7528009.464(-15743468.715-28349349.353)	433.960(-895.183-1620.105)	593851.260(-75101.570-1453125.529)	30.716(-4.817-76.079)	-8.36(-8.73,-7.98)	-92.11	-92.92
Low SDI	9040070.961(-23560542.479-33601994.658)	983.127(-2519.265-3604.095)	1838126.536(-3981598.847-6230665.126)	112.719(-236.399-376.092)	-7.17(-7.52,-6.82)	-79.67	-88.53
**Regions**							
Andean Latin America	35350.684(-40139.905-138602.837)	67.135(-73.046-259.137)	5496.881(2857.416-8836.070)	8.627(4.426-14.021)	-7.05(-7.34,-6.75)	-84.45	-87.15
Australasia	9.323(3.729-19.011)	0.060(0.024-0.122)	3.024(1.218-6.658)	0.016(0.006-0.034)	-3.20(-	-67.57	-73.28

					3.70, -2.69)		
Caribbean	66063.765(-157938.274-298506.477)	157.955(-373.620-707.947)	18742.260(-13006.891-63824.406)	47.733(-35.538-165.606)	-3.59(-4.00, -3.18)	-71.63	-69.78
Central Asia	43041.288(-49425.923-158747.554)	46.798(-51.007-169.817)	11074.408(862.426-22807.120)	11.316(1.045-23.155)	-5.24(-5.51, -4.97)	-74.27	-75.82
Central Europe	19799.662(1999.949-38064.255)	20.829(0.412-42.079)	2225.833(1175.812-3511.074)	3.787(1.894-6.182)	-5.41(-5.62, -5.21)	-88.76	-81.82
Central Latin America	182431.973(-306221.134-783071.071)	78.747(-130.455-335.760)	14047.932(2186.075-32348.054)	6.544(0.597-16.031)	-7.47(-7.96, -6.98)	-92.3	-91.69
Central Sub-Saharan Africa	688121.682(-2157010.795-2640388.853)	649.973(-1965.724-2451.004)	140785.218(-221128.050-411141.931)	70.702(-102.080-199.486)	-6.89(-7.55, -6.21)	-79.54	-89.12
East Asia	409401.737(-641549.087-1528734.202)	35.575(-56.171-133.356)	41003.640(25811.498-59891.346)	3.582(2.169-5.240)	-7.89(-8.61,	-89.98	-89.93

					-7.16)		
Eastern Europe	1211.982(195.249-2860.587)	0.714(0.070-1.733)	123.608(73.943-198.593)	0.107(0.064-0.171)	-6.51(-6.75,-6.27)	-89.8	-84.98
Eastern Sub-Saharan Africa	3576313.246(-8719060.527-13422344.039)	974.277(-2333.675-3604.419)	724403.903(-1501525.991-2539596.042)	115.129(-232.605-398.982)	-7.09(-7.34,-6.84)	-79.74	-88.18
High-income Asia Pacific	704.876(164.330-1460.560)	0.648(0.122-1.408)	77.217(12.698-193.070)	0.111(0.013-0.293)	-4.96(-5.33,-4.60)	-89.05	-82.86
High-income North America	478.684(112.480-1056.533)	0.222(0.053-0.488)	145.801(66.419-258.454)	0.066(0.030-0.119)	-4.08(-4.36,-3.80)	-69.54	-70.2
North Africa and Middle East	939526.208(-1781877.590-3559677.100)	184.694(-346.639-695.859)	136846.117(-88059.169-402316.887)	22.443(-15.335-67.156)	-7.90(-8.47,-7.33)	-85.43	-87.85
Oceania	19578.612(-36149.836-71242.430)	193.519(-349.849-698.928)	7522.777(-9520.357-26488.737)	39.465(-46.216-134.818)	-3.73(-4.26,	-61.58	-79.61

					-3.19)		
South Asia	6908283.315(-13657354.168-25551783.187)	443.311(-865.691-1628.790)	538590.310(116842.315-1191899.532)	32.333(5.934-74.173)	-8.14(-8.69,-7.58)	-92.2	-92.71
Southeast Asia	1687654.428(-4150863.978-6929318.765)	290.328(-713.283-1191.046)	91620.072(-23020.438-238795.046)	15.544(-5.106-42.214)	-9.14(-9.28,-8.99)	-94.57	-94.65
Southern Latin America	7562.336(-2770.465-19430.212)	14.755(-5.547-38.051)	1341.159(525.529-2838.557)	2.971(1.107-6.402)	-4.67(-4.88,-4.47)	-82.27	-79.86
Southern Sub-Saharan Africa	192794.766(-333745.469-789086.418)	257.999(-439.770-1048.952)	39503.316(-25380.938-139519.653)	49.392(-32.907-176.445)	-4.74(-5.14,-4.33)	-79.51	-80.86
Tropical Latin America	202676.034(-370115.378-799241.397)	122.195(-232.166-491.840)	20874.966(11273.311-32512.597)	11.298(5.887-17.837)	-7.81(-8.11,-7.52)	-89.7	-90.75
Western Europe	1814.292(738.669-3322.778)	0.765(0.299-1.430)	418.493(163.506-775.817)	0.188(0.068-0.356)	-4.00(-4.28,	-76.93	-75.44

					-3.72)		
Western Sub-Saharan Africa	3811953.420(-11479504.532-14516341.034)	1048.819(-3115.702-3958.693)	868908.735(-2286585.443-2975602.663)	110.332(-280.573-369.431)	-7.52(-7.92, -7.13)	-77.21	-89.48

Abbreviations: ASR, age-standardized rate; RC, relative change; EAPC, estimated annual percentage change; SDI, sociodemographic index; UI, uncertainty interval; CI, confidence interval.

Table S3 The Model Validation Results for ARIMA Forecasting.

Measure	Sex	p	d	q	AIC	BIC	Kpss_ Stat	Kpss_ Pval	Lb_Stat	Lb_Pval
Deaths	Male	0.00	2.00	1.00	-138.76	-135.96	0.89	0.01	12.10	0.21
	Female	0.00	2.00	1.00	-128.80	-126.00	0.88	0.01	11.91	0.22
	Both	0.00	2.00	1.00	-135.03	-132.23	0.89	0.01	11.74	0.23
DALYs	Male	0.00	2.00	0.00	-160.08	-158.68	0.89	0.01	14.91	0.14
	Female	0.00	2.00	0.00	-151.68	-150.28	0.89	0.01	13.04	0.22
	Both	0.00	2.00	0.00	-156.86	-155.45	0.89	0.01	13.95	0.18

Abbreviation: p: Partial Autocorrelation Order; d: Differencing Order; q: Moving Average Order; AIC: Akaike Information Criterion; BIC: Bayesian Information Criterion; Kpss_Stat: KPSS Statistic; Kpss_Pval: KPSS Test P-Value; Lb_Stat: Ljung-Box Statistic; Lb_Pval: Ljung-Box Test P-Value
